# Oncolytic adenovirus armed with IL-24 Inhibits the growth of breast cancer *in vitro* and *in vivo*

**DOI:** 10.1186/1756-9966-31-51

**Published:** 2012-05-28

**Authors:** Wei Zhu, Lai Wei, Hongwei Zhang, Junxue Chen, Xinyu Qin

**Affiliations:** 1 Department of General Surgery, Zhongshan Hospital, Fudan University, Shanghai, 200032, China; 2 Department of cardiac Surgery, Zhongshan Hospital, Fudan University, Shanghai, 200032, China

**Keywords:** Breast cancer, IL-24, Oncolytic adenovirus

## Abstract

**Background:**

Interleukin-24 (IL-24) is a cytokine that belongs to the IL-10 family. It can selectively induce cancer cell apoptosis which has been utilized as a cancer gene therapy strategy.

**Methods:**

A recombinant type five adenovirus containing IL-24 gene (designated CNHK600-IL24) was constructed, whose replication is activated only in tumor cells. The replication of CNHK600-IL24 in breast tumor cells and fibroblasts were assessed by TCID50 and MTT assay; the secretion of IL-24 was measured by ELISA and western blotting. The in vivo anti-tumor effect of CNHK600-IL24 was investigated in nude mice carrying orthotopic or metastatic breast tumor.

**Results:**

We observed that CNHK600-IL24 could replicate efficiently and resulted in high level IL-24 expression and massive cell death in human breast cancer cell MDA-MB-231 but not in normal fibroblast cell MRC-5. In addition, orthotopic breast tumor growth in the nude mice model was significantly suppressed when CNHK600-IL24 was administered. In the metastatic model generated by tail vein injection, CNHK600-IL24 virotherapy significantly improved survival compared with the same virus expressing EGFP (median survival CNHK600-IL24, 55 days vs. CNHK600-EGFP, 41 day, p < 0.05 Mantal-Cox test). A similar phenomenon was observed in the metastatic model achieved by left ventricular injection as suggested by in vivo luminescence imaging of tumor growth.

**Conclusion:**

The oncolytic adenovirus armed with IL-24, which exhibited enhanced anti-tumor activity and improved survival, is a promising candidate for virotherapy of breast cancer.

## Background

In women, breast cancer is the most frequently diagnosed malignant neoplasm and causes one of the highest mortality among all malignancies. Worldwide, over 1.3 million new cases of invasive breast cancer are diagnosed, and more than 450,000 women die from breast cancer annually [1]. Despite the advances made in the diagnosis and treatment of early breast cancer which has contributed to the declining mortality, metastatic breast cancer remains an incurable disease. More efficacious therapies to prevent relapse in early stage patients and to treat metastatic disease are needed.

Interleukin-24 (IL-24) is an important immune mediator, as well as a broad-spectrum tumor suppressor. Delivery of IL-24 by liposome or adenovirus can specifically inhibit growth of tumor cells and induce tumor-specific apoptosis [2-6]. Traditional replication-defective adenovirus cannot target tumor cells, which limits its therapeutic value. Replication selective virotherapy holds great promise for the treatment of cancer [7-9] whose appealing features include tumor-selective targeting, viral self-spreading in cancer cells, and no cross-resistance to current treatments. One strategy to achieve tumor specificity is the use of tumor- or tissue-specific promoters, such as MUC1, PSA, or PS2, to drive adenoviral genes that are essential for replication [10,11]. This system allows the oncolytic adenovirus to selectively replicate in tumor cells without affecting normal tissues [12]. Human telomerase reverse transcriptase (hTERT) is a catalytic subunit of telomerase and determines the activity of telomerase. The expression of hTERT is found in more than 85% of tumor cells, whereas it is absent from most normal cells [13]. Therapeutic genes under the control of the hTERT promoter will selectively express in telomerase-positive tumor cells at a high level [14]. In addition, in the progression of malignancy, uncontrolled proliferation of tumor cells often leads to a rapid increase in cellular oxygen consumption, resulting in a hypoxic microenvironment within the tumor, which is especially prominent in solid tumors. Hypoxic signaling in tumor cells induces the expression of hypoxia-inducible factor-1 (HIF-1) [15]. HIF-1 binds to the hypoxia response element (HRE) and activates the transcription of target genes. Therefore, the HRE promoter can be introduced to recombinant adenovirus to confine the oncolytic effect to hypoxic tumor cells. Combining these specific promoters into dual-promoter constructs will further enhance the targeting of virus and improve the safety of the treatment [16].

In this study, we used *hTERT* promoter to regulate the adenoviral *E1A* gene, HRE promoter to control the adenoviral *E1B* gene, and inserted the CMV promoter driven *IL-24* expression cassette between *E1A* and *E1B* which resulted in the oncolytic adenovirus CNHK600-IL24. We aimed to assess the antitumor selectivity and therapeutic potential of CNHK600-IL24 for breast cancer both *in vitro* and *in vivo*.

## Methods

### Cells and cell culture

Human embryonic kidney 293 (HEK293) cells were purchased from Microbix Biosystems. The human breast cancer cell line MDA-MB-231 and the normal fibroblast cell line MRC-5 were purchased from Shanghai Laboratory Animal Center, Chinese Academy of Sciences. HEK293 and MRC-5 cells were maintained in Eagle’s minimal essential medium (EMEM) supplemented with 10% fetal bovine serum (FBS), at 37°C, 5% CO_2._ MDA-MB-231 cells were cultured in Leibovitz’s L15 medium containing 10% FBS, at 37°C in CO_2_-free conditions.

### Construction and preparation of the oncolytic adenovirus CNHK600-IL24

The oncolytic adenovirus ZD55-IL24 was kindly provided by Professor Xin-yuan Liu from the Shanghai Institutes for Biological Sciences of the Chinese Academy of Sciences. Plasmid pXC1 was purchased from Microbix Biosystems Company, Canada. pClon9, pUC19-INS, SG502-△CR2 and the adenovirus backbone plasmid pPE3 were constructed by the Laboratory of Gene and Viral Therapy, Eastern Hepatobiliary Surgical Hospital, Second Military Medical University, Shanghai. Restriction enzymes were purchased from New England Biolabs.

Plasmid pCLON9 was digested with XhoI and SpeI, and pUC19-INS was digested with XbaI and SalI. The resulting 2680 bp and 1211 bp DNA fragments were ligated to create pCLON9-INS. The *IL-24* expression cassette includes the human cytomegalovirus (hCMV) immediate-early promoter, the *IL-24* gene and the SV40 PolyA sequence. It was extracted from ZD55-IL24 by BglII digestion and inserted into pCLON9-INS, which was digested with BamHI. The recombinant product was named pCLON9-INS-IL24 and sent to Shanghai GeneCore Biotechnologies Co. Ltd. for sequencing. After digestion with AgeI and NotI, SG502-ΔCR2 and pCLON9-INS-IL24 were ligated to form SG502-INS-IL24. To obtain the virus, the plasmid SG502-INS-IL24 and type 5 adenovirus pPE3 were cotransfected into HEK293 cells with Lipofectamine 2000 (GIBCO BRL). The recombinant virus was verified by repeated PCR amplification. The correct recombinant virus, named CNHK600-IL24, was amplified in 293 cells and purified by cesium chloride density gradient centrifugation. Oncolytic adenovirus CNHK600-EGFP, which carries enhanced green fluorescent protein (EGFP) as a reporter gene, was constructed and prepared in the same way. Median tissue culture infective dose method (TCID50) was used to determine the virus titer.

### Fluorescence microscopy

MDA-MB-231 cells and MRC-5 cells were infected with CNHK600-EGFP at a multiplicity of infection (MOI) of 1 and observed under the fluorescence microscope. Photographs were taken 48 h, 72 h and 96 h after infection.

### Viral replication assay

Logarithmic phase MDA-MB-231 and MRC-5 cells were seeded at 1 × 10^5^ cells/ml into 6-well plates. The cells were infected with CNHK600-IL24 and CNHK600-EGFP at MOI of 5. Two hours after incubation with the viruses, the supernatants were discarded and replaced with 3 ml culture medium containing 5% FBS. At timepoints 0, 12, 24, 48, 72 and 96 hours after infection, the cells were scraped and transferred to five-ml centrifuge tubes and underwent three cycles of freezing and thawing between 37°C and −80°C. The TCID50 method was used to determine titre.

### Cell growth inhibition assay

Log phase MDA-MB-231 cells and MRC-5 cells were adjusted to 1 × 10^5^ cells/ml with culture medium containing 10% FBS, and 100 μl/well was added to 96-well plates. The cells were incubated at 37°C for 18 h and then infected with CNHK600-IL24 and CNHK600-EGFP at MOI values of 0, 0.1, 0.5, 1, 5, 10, 100 and 1000. Two hours after incubation with virus, the supernatants were discarded and replaced with 100 μl culture medium containing 5% FBS. Five days after infection, 100 μl 3-(4, 5-Dimethylthiazol-2-yl)-2,5-diphenyltetrazolium bromide (MTT, Sigma-Aldrich) at 1 mg/ml was added. The plates were incubated at 37°C for 4 h, and then the supernatants were discarded and 100 μl DMSO (Merker) was added. After 15 min shaking, absorbances at 490 nm were measured.

### Detection of IL-24 protein in culture supernatants and cells

Log phase MDA-MB-231 and MRC-5 cells were adjusted to 1 × 10^5^ cells/ml and added to 6-well plates. The cells were infected with CNHK600-IL24 at a MOI of 5. Two hours after incubation, the medium was replaced with fresh culture medium supplemented with 5% FBS. Supernatants were collected at 12, 24, 48 and 96 h after infection. The expression of IL-24 was measured with a standard ELISA assay (GBD Biosciences Catalog No. I083). At the same time, cells were lysed on ice with 500 μl lysis buffer (10 mM Tris-Cl, pH 7.4, 0.15 M NaCl, 5 mM EDTA, 1% Triton X100, 5 mM DTT, 0.1 mM PMSF, 5 mM ε-aminocaproic acid) per well. The cell lysates were centrifuged at 10,000 g, 4°C for 10 min, and then the supernatants were stored at −80°C until used for western blotting to detect the expression of IL-24 protein.

### Establishment and treatment of the orthotopic breast cancer model in nude mice

*Nu/nu* female mice, aged 5- to 6-weeks old and weighing about 18 to 20 g, were cultivated by the Shanghai Experimental Animal Center of Chinese Academy of Sciences. All procedures were approved by the Committee on the Use and Care on Animals and done in accordance with the institution guidelines. Log phase MDA-MB-231-luc cells (Xenogen Corporation) were diluted with sterile PBS to 8 × 10^7^ cells/ml and mixed with matrigel at a 1:1 ratio. After inhalation anesthesia, 50 μl cells were injected into the fat pad of nude mice. At timepoints 14, 16, 18, 20 and 22 days after the injection of cells, viruses were administered through intravenous injection. Fifteen nude mice were divided into groups as follows: each mouse in the control group was injected with 100 μl saline, the CNHK600-EGFP group was injected with 2 × 10^8^ pfu virus (100 μl), the low-dose group of CNHK600-IL24 received 1 × 10^8^ pfu virus (100 μl), the medium-dose group of CNHK600-IL24 received 2 × 10^8^ pfu (100 μl), and the high-dose group of CNHK600-IL24 received 4 × 10^8^ pfu (100 μl). Bioluminescence was measured weekly using an in vivo imaging system (IVIS 50, Xenogen Corporation). On day 42, mice were sacrificed after anesthesia and the tumors were separated, weighed and fixed in 4% formaldehyde. The tumor inhibition rate was calculated according to the following formula: Tumor Inhibition Rate = (mean of tumor weight in control group - mean of tumor weight in treatment group)/mean of tumor weight in control group × 100%.

### Immunohistochemistry and *in situ* TUNNEL assay

Immunohistochemical analysis of hexon (GENWAYBIO) and IL-24 (USCN LIFE, USA) was performed on paraffin sections. Briefly, sections were deparaffinized in xylene, hydrated through graded alcohols and water, endogenous peroxidases were inactivated with 3% hydrogen peroxide in phosphate-buffered saline (PBS) followed by incubation with the primary antibody for one hour at room temperature and with the biotinylated secondary antibody (anti-mouse IgG) for 1 hour. After incubation with streatavidin-HRP for 10 minutes, sections were washed and developed with DAB substrate for 3–10 minutes. For in situ TUNEL (Keygen Bio-Technology Development Co., Ltd. Nanjing, China) assay, sections were deparaffinized and hydrated as described above. After proteinase K digestion, Terminal deoxynucleotidyl transferase (TdT) and dUTP-biotin was applied for 1hour at 37°C. After washing with PBS, sections were incubated with streptavidin-HRP and developed with DAB for 10 min.

### Establishment and treatment of metastatic model of breast tumor

We used two models of metastatic breast cancer using tail vein injection and left ventricular injection of MDA-MB-231-luc cells. In the first model, MDA-MB-231-luc cells was adjusted to 1 × 10^6^ cells/ml, and 100 μl was intravenously injected into nude mice after inhalation anesthesia. Viruses were intravenously administrated on days 10, 12, 14, 16 and 18 after cell injection. Twenty-four nude mice were evenly divided into three groups: each mouse in the control group was injected with 150 μl saline, and each mouse in the CNHK600-EGFP and CNHK600-IL24 groups received 4 × 10^8^ pfu of the appropriate virus (150 μl). In vivo imaging of tumors was performed using IVIS 50 on day 0, 10, 17, 24, 31 and 38. The survival time of mice in each group was recorded and plotted for survival curves. In the second model, the same amount of MDA-MB-231-luc cells were used and injected into the left heart ventricle after inhalation anesthesia, followed by immediate imaging to determine if the modeling was successful. Six mice with successfully established metastases were divided into two groups. The control mouse was injected with 150 μl saline, and the mice in the CNHK600-IL24 group were injected with 4 × 10^8^pfu (150 μl) of virus, administrated through the tail vein on days 10, 12, 14, 16 and 18. In vivo imaging of tumors was performed using IVIS 50 on days 0, 10, 17, 24, 31, 38 and 45. On day 45, mice were sacrificed after anesthesia, and organs were separated, immersed immediately in fluorescein (300 μg/ml) and tested for bioluminescence ex vivo.

### Statistical analysis

The experimental data are presented as mean ± SD. All statistical analyses were performed with the Statistical Product and Service Solutions 12.0 (SPSS Inc., Chicago, USA) and Prism 5 (Praphpad, USA) software. Student’s *t*-test and one-way ANOVA analyses were employed to compare two groups and multiple groups respectively. Survival curves were plotted according to the Kaplan-Meier method and log-rank test was used to compare survival of mice receiving different therapies. Data were considered statistically significant when p < 0.05.

## Results

### Oncolytic activity of CNHK600-IL24 in vitro

**Figure 1:**
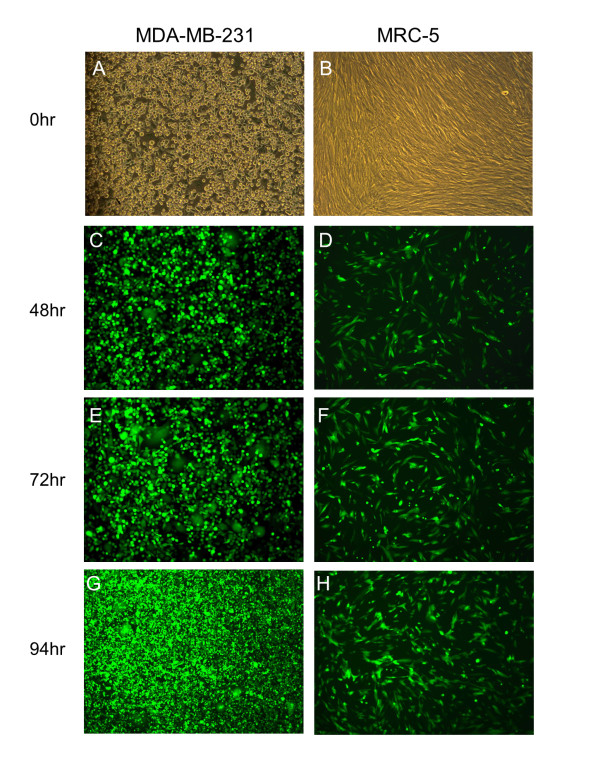
**The proliferation of oncolytic adenovirus expressive EGFP.** The MDA-MB-231 cells (**A**) and MRC-5 cells (**B**) were infected with CNHK600-EGFP at a MOI of 1. The viral replication was monitored under the fluorescence microscope at 48 hr (**C**, **D**), 72 hr (**E**, **F**) and 96 hr (**G**, **H**) after infection.

**Figure 2:**
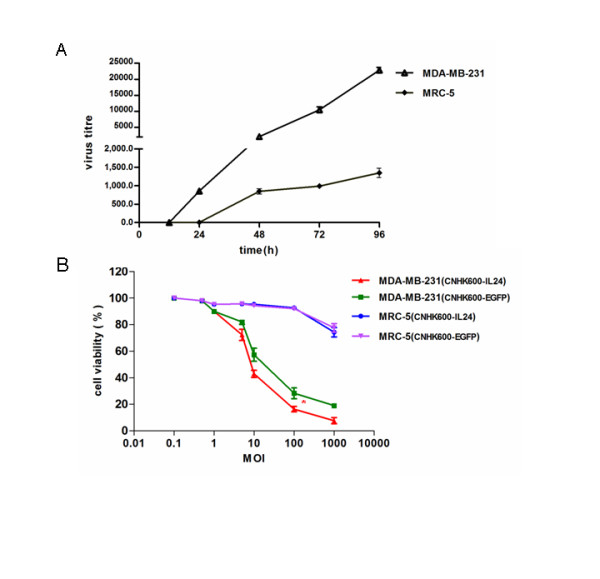
**CNHK600-IL24 selectively produced IL-24 and induced cell death in a breast cancer cell line.** (**A**) Selective replication of CNHK600-IL24 in MDA-MB-231 cells. MRC-5 or MDA-MB-231 cells were infected with CNHK600-IL24 at a MOI of 5 and virus titers from the supernatant were measured by the TCID50 method at indicated time points. (**B**) Five days after infection with CNHK600-IL24 or CNHK600-EGFP at the indicated range of MOI, the viability of MDA-MB-231 and MRC-5 was measured by MTT assay.

We constructed the adenovirus containing IL-24 gene, namely CNHK600-IL24, as described in the material and method. The titer of CNHK600-IL24 after amplification and purification was 1.9 × 10^10^ pfu/ml. The titer of CNHK600-EGFP was 1.1 × 10^10^ pfu/ml. In order to test the selective propagation of the recombinant virus, we first observed the growth characteristics of the oncolytic adenovirus expressing EGFP in malignant and normal cells. After infection with CNHK600-EGFP, the expression of green fluorescence in MDA-MB-231 cells was initially scattered and gradually turned into a widespread, centralized and integrated presence, indicating that the virus proliferated efficiently in breast cancer cells. In contrast, only sparse fluorescence was observed in normal fibroblast cells (MRC-5) after CNHK600-EGFP infection, indicating no significant viral proliferation (Figure 1). The growth curve of CNHK600-IL24 in MDA-MB-231 and MRC-5 cells were also measured. As shown in Figure 2A, at 48 hours after infection, the proliferation rate of CNHK600-IL24 in breast cancer cells was significantly accelerated. The viral load was over 10,000 fold higher at 72 h, and 20,000 fold at 96 h post-infection. In contrast, proliferation of the virus in MRC-5 was not significant; the viral load was only 1000 fold higher at 72 h and 96 h post-infection (Figure 2A). The proliferation of CNHK600-EGFP in MDA-MB-231 and MRC-5 was similar to that of CNHK600-IL24 (data not shown).

**Figure 3:**
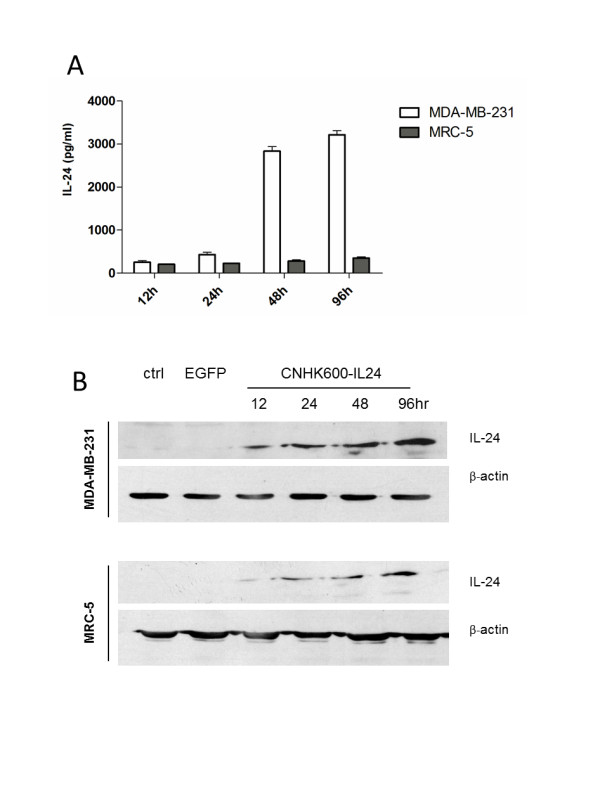
**Expression of IL-24 in MDA-MB-231cells and MRC-5 cells.** (**A**) The concentration of IL-24 in the supernatant after infection of CNHK600-IL24, as measured by ELISA. (**B**) Relative quantification of IL-24 by western blotting, the expression of β-actin was measured as loading control.

Next, we assessed the selective killing of CNHK600-IL24 on malignant tumor cells. As shown in Figure 2B, at a MOI of 10, CNHK600-IL24 killed 57% of the breast cancer MDA-MB-231 cells. At a MOI of 100, only 16% of the cancer cells survived. In contrast, 94% of MRC-5 cells survived at a MOI of 100 of CNHK600-IL24. The impact of CNHK600-EGFP on MDA-MB-231 cell survival was weaker than that of CNHK600-IL24, at the same MOI of 100pfu/cell, 28.3% of the cancer cells survived after the infection of CNHK600-EGFP whereas only 16.3% remained viable after CNHK600-IL24 infection (Figure 2B, p < 0.05 student’s *t*-test). This suggested that expression of IL-24 enhanced the oncolytic activity of adenovirus. The expression of IL-24 in breast cancer cells and normal fibroblast was quantified by ELISA and western blotting assays. As expected, 48 hours after infection of CNHK600-IL24, the concentration of IL-24 protein in supernatants of infected breast cancer cells was significantly elevated (3 ng/ml), whereas the level of IL-24 MRC-5 cells remained low (Figure 3A). Similarly, the expression of IL-24 protein in the lysates of breast cancer cells was significantly increased, whereas the IL-24 levels in normal fibroblasts remained difficult to detect (Figure 3B).

### CNHK600-IL24 inhibited orthotopic breast tumor growth and tumor metastasis in vivo

**Figure 4:**
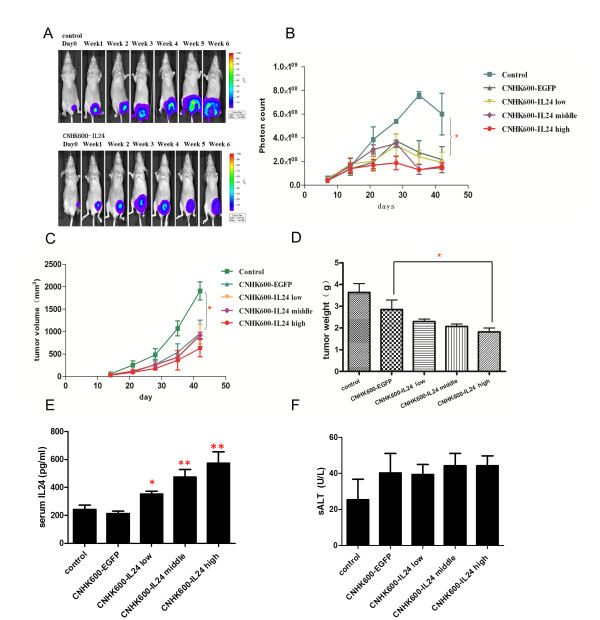
**Suppression of the tumor in nude mice bearing orthotopic breast cancer after CNHK600-EGFP or CNHK600-IL24 was injected by tail vein.** Log phase MDA-MB-231-luc cells were injected into the fat pad of nude mice. At 14, 16, 18, 20 and 22 days after the injection of cells, viruses were administered through intravenous injection at the dose of 2 × 10^8^ pfu (CNHK600-EGFP and CNHK600-IL24 middle). The doses for CNHK600-IL24 low and high group were 1× 10^8^ and 4× 10^8^ pfu respectively. Luminescent images were visualized every week (**A**), Photon counts (**B**) and tumor volume (**C**) were also measured. Mice were sacrificed and tumor weight was measured on day 42 (**D**). Mouse serum was collected on day 42 after orthotopic tumor cell inoculation. IL24 level was measured by ELISA (**E**) and serum ALT level was also quantified (**F**) (N = 5 for each group).

Having established the oncolytic property of CNHK600-IL24 virus, we next investigated its anti-tumor activity in mice models. We first established an orthotopic breast tumor model in nude mice and the growth of tumor can be visualized by live luminescence imaging. After injection of breast cancer cells, the tumors were detected weekly with IVIS 50 (Figure 4A), and the photon counts were measured. As illustrated in Figure 4B, the number of photons in CNHK600-EGFP and CNHK600-IL24 groups were significantly lower than that of the control group (one-way ANOVA, P < 0.05). Fourteen days after injection, the tumors in all of the mice were palpable. The growth curves of the tumors in each group are plotted according to weekly measurements of tumor sizes (Figure 4C). The tumor volumes of mice in the control group were significantly greater than those of the CNHK600-EGFP and CNHK600-IL24 groups (one-way ANOVA, *P* <0.05).

**Figure 5:**
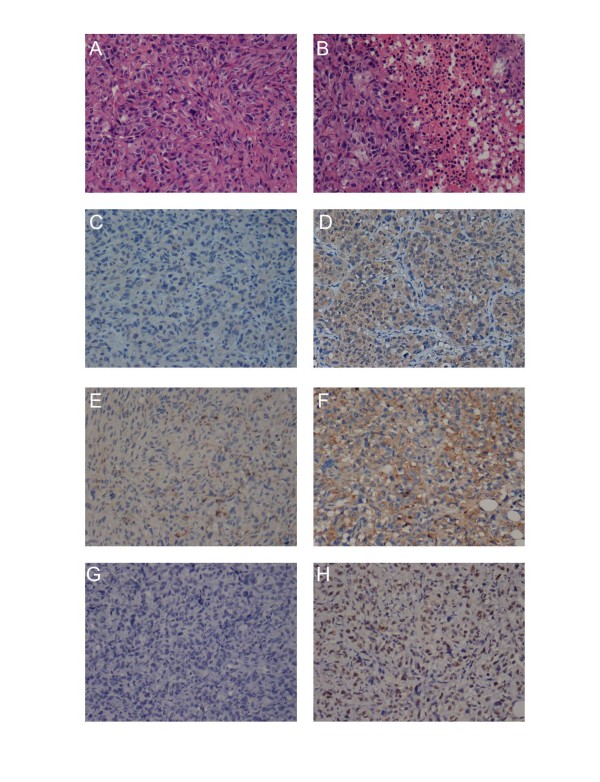
**Histopathology and immunohistochemistry of tumor tissues with CNHK600-IL24 treatment.** HE staining of tumor tissue in the control group (**A**) and in CNHK600-IL24 treatment group (**B**) was visualized. The expression of adenovirus hexon protein (**C**, **D**) and IL-24 (**E**, **F**) were monitored by immunohistochemistry. Breast tumor cell apoptosis were measured by TUNEL assay (**G**, **H**).

Mice were sacrificed after anesthesia on day 42, and the tumors were separated and weighed (Figure 4D). In CNHK600-EGFP group, the tumor inhibition rate was 21.49%, and the tumor inhibition rates of the CNHK600-IL24 low-dose, medium-dose and high-dose groups reached 36.91%, 42.98% and 49.86%, respectively (P < 0.05, EGFP group vs. IL24 high-dose group student’s *t*-test). In addition, we assessed the level of secreted IL24 in mouse serum. As shown in Figure 4E, injection of CNHK600-IL24 in all three dosage schemes caused significant elevation of serum IL24 compared with control group(p < 0.05 in low dose, p < 0.01 in middle and high dose) which was further confirmed by immunohistochemical staining (see below). To examine potential side-effects caused by adenovirus infection, we measured serum ALT levels after treatment. A slight elevation in ALT indicated that our tumor specific adenovirus did not cause pronounced liver toxicity (Figure 4F). HE staining revealed apparent tumor necrosis in CNHK600-IL24 treatment group (Figure 5A, B). Immunohistochemical assays showed that the expression of IL-24 protein and the adenovirus capsid protein hexon were positive in the CNHK600-IL24 treatment group but negative in the control group (Figure 5C, D, E, F). TUNEL assay was utilized to measure apoptosis in tumors. As shown in Figure 5G, 5H, the level of apoptosis in the CNHK600-IL24 treated tumors was significant, whereas the level of apoptosis in the control group was negligible.

**Figure 6:**
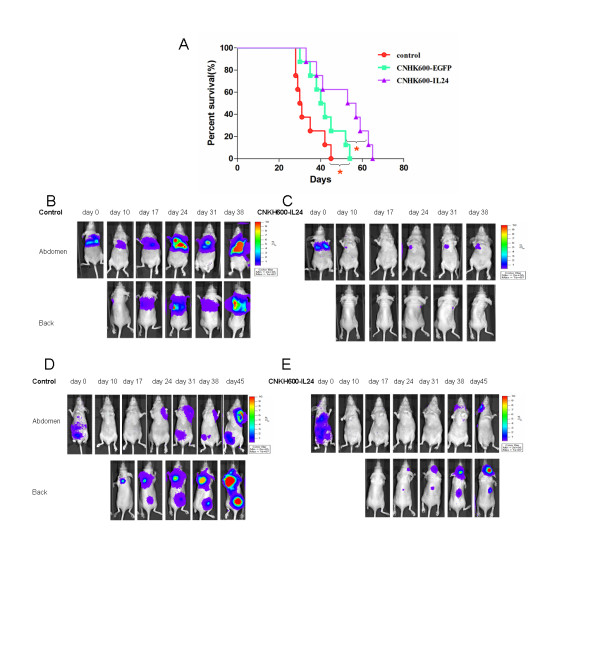
**Inhibition of breast tumor metastasis by CNHK600-IL24.** (**A**) Survival curves of mice in the metastatic model created by tail vein injection of cancer cells. (N = 8 for each group) (**B**, **C**) In vivo imaging of the control and the CNHK600-IL24 group in the metastatic model created by tail vein injection. (**D**, **E**) In vivo imaging of the control and CNHK600-IL24 group in the metastatic model generated by left ventricular injection.

We next examined whether CNHK600-IL24 can effectively reduce breast tumor metastasis in a tail vein injection model in nude mice. As shown in the Kaplan-Meier plot (Figure 6A), the median survival in the control group was 30.5 days, whereas injection of the oncolytic adenovirus significantly prolong the survival time (CNHK600-EGFP, 41 day, p < 0.05 and CNHK600-IL24, 55 days, p < 0.01, Mantal-Cox test). When comparing CNHK600-EGFP with CNHK600-IL24, a significant difference was also observed (p = 0.0447, Mantal-Cox test). We further monitored the growth of the metastatic tumor foci by in vivo imaging (Figure 6B, 6C). Indeed, the ascending luminescence signal as observed in the control mice was well suppressed in the CNHK600-IL24 group.

We also assessed the anti-proliferative activity of CNHK600-IL24 in a metastatic model by left ventricular injection. Similarly, two of the three mice in control group died on days 36 and 41, but the three CNHK600-IL24-treated mice all survived more than 45 days. From the 10th day on, all of the mice were tested using IVIS 50 every seven days. There was an obvious difference in metastases between the control group and treatment group (Figure 6D, 6E). On day 45, surviving mice were sacrificed and the metastases were detected ex vivo. There were extensive metastases in the only surviving mouse of the control group. Tumors were visible in the skull, mandible, scapula, clavicle, femur, brain, lung and liver. In contrast, metastases in the treatment groups were significantly reduced (data not show).

## Discussion

Breast cancer is the most frequently diagnosed neoplasm in women. Although great progress has been made in treatment of breast cancer, very limited options are available for metastatic breast cancer. Indeed, micrometastases within bone marrow or other tissues can lead to relapse and metastasis and significantly accelerate the progression of disease[17]. Targeted oncolytic adenovirus brought new options for novel strategies to tackle these difficult problems.

Compared with small molecule drug or recombinant proteins, viruses have their unique properties, i.e., they can replicate and spread in addition to carrying anti-tumoral therapeutic genes, and may be targeted specifically to tumor cells. In recent years, the synergistic anti-tumor effects of IL-24, including apoptosis-inducing and immune-stimulating effects have gained increasing attention. Zheng et al. found that Adenovirus transduction of IL-24 causes G2/M cell cycle arrest and apoptotic cell death and this effect could be antagonized by IL-10[18]. Patani et al. showed that recombinant IL-24 reduced the motility and migration of MDA-MB-231 using wound healing and electric cell impedance sensing assay. Furthermore, significantly lower expression of IL-24 was also noted in tumors from patients who died of breast cancer compared to those who remained disease free. Low levels of MDA-7 were significantly correlated with a shorter disease free survival[19]. Indeed, Introgen therapeutics has developed INGN241, a replication-defective adenovirus carrying IL-24, which has shown satisfactory efficacy and safety during phase I and phase II clinical trials [20,21]. Sarkar *et al.* constructed Ad.PEG-E1A-IL24 in which *E1A* was under the control of PEG-3 promoter. In their study, breast cancer cell line T47D cells were implanted subcutaneously in nude mice to establish animal models, and the recombinant adenovirus was injected intratumorally. Four weeks after administration, all tumors were eliminated, including the contralateral abdominal metastases [22]. In theory, the dual-regulated oncolytic adenovirus has better safety and targeting and thus is more suitable for clinical treatment of cancer [23].

In this study, we constructed CNHK600-IL24, which was regulated by both the hTERT and HRE promoters and was armed with the *IL-24* gene. Our replication selective vector design is much more advantageous compared with replication defective adenoviruses as previous experience has indicated that the latter type cannot specifically target cancer cells. The *EGFP* gene was inserted at the same position instead of *IL-24* in CNHK600-EGFP to facilitate the observation of virus proliferation under the fluorescence microscope. Results showed that CNHK600-EGFP replicated rapidly in tumor cells and expressed the exogenous gene efficiently, which was further verified by virus proliferation assay. In addition, in *vitro* experiments confirmed that CNHK600-IL24 proliferated specifically in breast cancer cells and selectively killed tumor cells.

To evaluate the effects of CNHK600-IL24 in vivo, we established an orthotopic breast cancer model by injecting cells from the breast cancer cell line MDA-MB-231 harboring a luciferase gene (luc) into the mammary fat pads of nude mice. Two metastatic models of breast cancer were established by intravenous and left-ventricular injection of tumor cells. An in vivo optical imaging system was applied to observe the inhibitory effect of the CNHK600-IL24 adenovirus on breast cancer *in vivo*. In vivo optical imaging technology allows continuous observation of the same group of animals, which results in more significant and reliable data [24].

In the orthotopic breast cancer model in nude mice, the results of *in vivo* imaging showed that the number of photons in the CNHK600-EGFP group and the CNHK600-IL24 treatment group were significantly lower than those of the control group. The tumor volumes of the CNHK600-EGFP group and the CNHK600-IL24 treatment group were also significantly smaller, demonstrating the potent anti-tumor effects of the oncolytic adenovirus CNHK600-IL24. Large areas of necrosis in tumor tissue were found by pathological assay, which possibly resulted from continuous replication of the oncolytic adenovirus and the ultimate lysis of tumor cells. Immunohistochemical analysis showed that in the CNHK600-IL24 treatment group, tumor cells were strongly positive for the adenoviral capsid protein hexon, whereas those of the control group were negative. This illustrates that after injection of CNHK600-IL24 through the tail vein, the virus reached the tumor and effectively replicated in the tumor cells.

In the metastatic model by tail vein injection, there was intense luminescence in the lungs of the control mice, but the photon intensity in the CNHK600-IL24 treated mice was significantly weakened. The survival time of mice in control group was significantly shorter than that of the CNHK600-EGFP and CNHK600-IL24 groups. Furthermore, tumor-bearing mice in CNHK600-IL24 group survived longer than those of the CNHK600-EGFP group, indicating that the gene-virotherapy was more effective than virotherapy alone. Similarly, in the metastatic model by left ventricular injection, the intensity of fluorescence in treatment groups was significantly weaker than that of the control group. In addition, ex vivo imaging showed reduced metastases in CNHK600-IL24 treated mice.

## Conclusions

Our *in vitro* and *in vivo* observations demonstrated that oncolytic adenovirus expressing IL-24 can actively destroy breast tumor and significantly prolong survival. We hope that this targeting gene-virotherapy will provide a promising strategy for breast cancer treatment in combination with chemotherapy or other therapeutic modalities in the future.

## Competing interests

The authors declare that they have no competing interests.

## Authors’ contributions

WZ, LW, HZ and JC performed the experiments. WZ drafted the manuscript. XQ supervised the experimental work. All authors read and approved the final manuscript.
